# The bug in a teacup—monitoring arthropod–plant associations with environmental DNA from dried plant material

**DOI:** 10.1098/rsbl.2022.0091

**Published:** 2022-06-15

**Authors:** Henrik Krehenwinkel, Sven Weber, Sven Künzel, Susan R. Kennedy

**Affiliations:** ^1^ Trier University, Trier, Germany; ^2^ Max Planck Institute for Evolutionary Biology, Ploen, Germany

**Keywords:** eDNA, plant–arthropod interaction, arthropods, biomonitoring

## Abstract

Environmental DNA analysis (eDNA) has revolutionized the field of biomonitoring in the past years. Various sources have been shown to contain eDNA of diverse organisms, for example, water, soil, gut content and plant surfaces. Here we show that dried plant material is a highly promising source for arthropod community eDNA. We designed a metabarcoding assay to enrich diverse arthropod communities while preventing amplification of plant DNA. Using this assay, we analysed various commercially produced teas and herbs. These samples recovered ecologically and taxonomically diverse arthropod communities, a total of over a thousand species in more than 20 orders, many of them specific to their host plant and its geographical origin. Atypically for eDNA, arthropod DNA in dried plants shows very high temporal stability, opening up plant archives as a source for historical arthropod eDNA. Considering these results, dried plant material appears excellently suited as a novel tool to monitor arthropods and arthropod–plant interactions, detect agricultural pests and identify the geographical origin of imported plant material. The simplicity of our approach and the ability to detect highly diverse arthropod communities from all over the world in tea bags also highlights its utility for outreach purposes and to raise awareness about biodiversity.

## 1. Background

Arthropods, the most diverse animal group, are central to the function of global ecosystems as pollinators, prey and biocontrol agents, yet also include pest and parasite species of great economic and medical relevance [1]. Monitoring arthropod communities is essential to understand their diversity and interactions, and in light of recent reports on insect decline, has become a research priority [2]. Arthropods are often monitored by passive sampling, for example with Malaise or pitfall traps [2], but such methods have disadvantages. Different trapping methods often capture only a subset of arthropod diversity. Moreover, passive trapping is usually lethal and provides no information on arthropod–plant interactions, which are critical for understanding ecology and inferring consequences of species losses or invasions [3].

Recently, environmental DNA (eDNA) has been suggested as an alternative to traditional arthropod monitoring [4]. eDNA analysis has revolutionized the field of biomonitoring [5], and various substrates have been shown to contain arthropod eDNA [6]. A particularly promising source is the surface of plant material, on which arthropods deposit eDNA, for example in chew marks or faeces. These traces can be enriched by amplification from filtered surface washes of flowers, leaves or fruits [4,7].

While this approach provides insights into plant–arthropod associations, it also has shortcomings. eDNA on plant material has a short half-life and is quickly degraded by UV light or washed off by rain [8]. Also, surface washes recover arthropods primarily from the outside of plants, missing the diverse community of mining and galling arthropods contained within the plant tissue [9,10]. An approach that recovers arthropods from both outside and inside plants is the extraction of DNA from pulverized plant material. To facilitate the grinding process, plant material is usually dried after collection. This confers an important advantage: in a dried state, DNA is stable and suitable for long-term storage [11].

Here we explore whether dried, ground plant material can be used to recover plant-associated arthropod eDNA with long-term temporal stability. We design a novel metabarcoding assay to selectively enrich DNA of arthropods from extractions of homogenized plants while preventing amplification of plant DNA. Using this assay, we test the utility of dried plant material as a simple arthropod monitoring tool. We focus on a type of dried plant material found in many households: namely, teas and herbs. These ubiquitous products, which are often stored for long periods at room temperature, offer an ideal test for the efficacy of our protocol and the stability of dried eDNA. Furthermore, teas and herbs originate from a variety of plant taxa cultivated in different regions across the globe, and may thus allow the identification of host plant-specific arthropod communities and pinpoint the geographical origins of samples. Due to their economic importance, these plants generally have well characterized pest communities, providing a solid baseline to test the accurate recovery of plant–arthropod associations.

## 2. Methods

### (a) Samples, primer design, sequencing and sequence analysis

In DNA extracts of homogenized plants, arthropod DNA is expected to be greatly underrepresented relative to plant DNA. Therefore, primers have to be used which exclude the plant from amplification, while amplifying a broad range of arthropod taxa. Using an alignment of arthropods, other animals, fungi and plants (see electronic supplementary material), we identified a sequence stretch in the 3’-end of the COI barcode region that distinguishes plants from arthropods by three nucleotide mismatches (AAG in most arthropods, TTC in plants). This leads to 3’ G-G, A-A, A-A mismatches, which almost entirely suppress plant amplification [12,13]. The resulting primer is very broadly compatible across the arthropod tree of life, missing only a few spider families [14]. We combined this forward primer, *NoPlantF_270* (a modified version of [15], with two reverse primers: (1) a slightly modified version of the reverse primer *mlCOIintR* [16] to amplify a fragment of 116 bp, and (2) the barcoding primer *FoldegenRev* [17] for a fragment of 456 bp. We also used the published primer set *ZBJ-ArtF1c*/*ZBJ-ArtR2c* [18], which amplifies a fragment of 211 bp. This primer set was designed to amplify arthropod DNA from bat faeces and also excludes plants (table 1).

**Table 1.** Primers used. Start and end positions are based on the COI gene of the mitochondrial reference genome of *Drosophila melanogaster*. Fragment length includes primer-binding sequences. Primer combinations are hereafter referred to as A, B & C for clarity.

primer combination	Fw	Fw 5′–3′	Rv	Rv 5′–3′	start-end	fragment length
*A*	NoPlantF_270	RGCHTTYCCHCGWATAAAYAAYATAAG	mlCOIintR_W	GRGGRTAWACWGTTCAWCCWGTNCC	270–385	116
*B*	ZBJ-ArtF1c	AGATATTGGAACWTTATATTTTATTTTTGG	ZBJ-ArtR2c	WACTAATCAATTWCCAAATCCTCC	33–243	211
*C*	NoPlantF_270	RGCHTTYCCHCGWATAAAYAAYATAAG	Fol-degen-rev	TANACYTCNGGRTGNCCRAARAAYCA	270–725	456

We first tested the three primer combinations in a selection of three commercially available tea samples: two green teas (*Camellia sinensis*) and one dandelion tea (*Taraxacum* sp*.*). Two additional samples of wild-collected and powdered European beech (*Fagus sylvatica*) leaves were included. These samples are collected under standardized conditions as part of a Germany-wide biomonitoring effort [19]. Our aim was to identify the proportion of arthropod sequences in relation to sequences of non-target organisms, such as plants and fungi, and to compare the recovery of arthropod diversity by the three different primer combinations. Unlike typical eDNA samples, we did not wash the plant material and filter the DNA particles, but extracted DNA directly from the shredded plant material. Total DNA was isolated from 100 mg of plant sample from tea bags using a CTAB protocol (OPS Diagnostics, Lebanon, USA).

PCR amplification, library preparation, sequencing and sequence analysis were performed as described in de Kerdrel *et al*. [20]. In brief, all samples were amplified for the three primer combinations with 35 cycles at an annealing temperature of 46°C using the Qiagen Multiplex Kit according to the manufacturer's protocols (Qiagen Hilden, Germany). PCR products were then dual indexed and sequenced on an Illumina MiSeq with 2 × 300 bp reads (Illumina, San Diego, USA). Template-free and blank extraction control PCRs were sequenced alongside all samples. Demultiplexed reads were merged using PEAR [21] and quality filtered using the fastX Toolkit [22] for 90% of bases > Q30. The quality-filtered reads were dereplicated and clustered into 3% radius OTUs in USEARCH with a minimum size of three [23]. Only OTUs with a length of 64 bp, 157 bp and 403 bp after primer trimming were retained. Taxonomy was assigned to OTUs using BLASTn [24] against the whole NCBI database. A minimum similarity of 85% was used to assign an OTU to phylum and a minimum of 98% to assign family, genus and species. For OTUs with assigned species status and minimum coverage of 100 reads, information on ecology and distribution was compiled by web searches. An OTU table was built for the resulting arthropod dataset using USEARCH. Alpha and beta diversity patterns were analysed using *vegan* [25] in R [26].

Based on the previous analysis, we identified primer combination A as most promising for the recovery of arthropod diversity from plant material. To explore the utility of our assay in more detail, we analysed a larger set of commercial teas and herbs, all purchased in local grocery stores in Trier, Germany. We included a total of 40 samples from four plants (*N*_Chamomile_ = 11, *N*_Mint_ = 10, *N*_Tea_ = 12, *N*_Parsley_ = 7), which belonged to a total of 17 brands (electronic supplementary material, table S1). Between one and four replicate extractions from different tea bags of the same product were included for most samples. The samples were processed as described above.

## 3. Results

**Figure 1.**
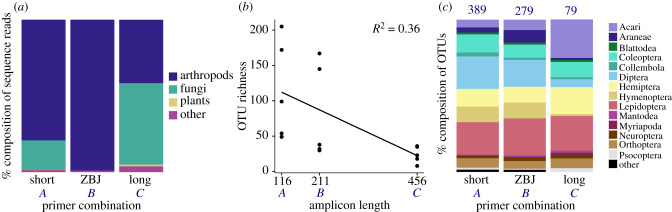
Comparison of the three primer combinations. (*a*) Barplot showing per cent composition of OTUs representing different phyla. (*b*) Scatterplot showing recovered taxonomic richness by amplicon length obtained from each primer set. (*c*) Barplot showing per cent composition of OTUs by arthropod order. Numbers above bars indicate total number of OTUs recovered by each primer combination. Letters *A*, *B* and *C* on *x*-axis correspond to primer combinations referred to in text; table 1 for details.

All three primer pairs were highly efficient at blocking plant amplification (combination *A* = 0.09%, *B* = 0.00%, *C* = 1.15% plant sequences; table 1 for primer combinations). We found a significant difference in arthropod recovery between the markers (combination *A* = 79.19%, *B* = 99.00%, *C* = 41.64% arthropod reads; pairwise Wilcoxon test, *p* < 0.05). For combinations *A* and *C*, fungi were particularly abundant (*A* = 19.40%, *C* = 53.39%; figure 1*a*). A significant negative correlation was found between amplicon length and OTU richness (figure 1*b*; *R*^2^ = 0.36, LM, *p* < 0.05). This effect was particularly evident for the longest amplicon, which only recovered about one fifth of the richness of the shortest one (total OTUs *A* = 421, *B* = 301, *C* = 87). While combinations *A* and *B* recovered comparable order compositions, the composition was significantly biased toward mites for the long amplicon C (*A* = 5.75%, *B* = 6.31%, *C* = 22.99% mite OTUs; pairwise Wilcoxon test, *p* < 0.05). All amplicons provided good taxonomic resolution, with no significant difference in species assignment success (63% of OTUs could be classified to species for each marker; Fisher's exact test *p* > 0.05).

**Figure 2.**
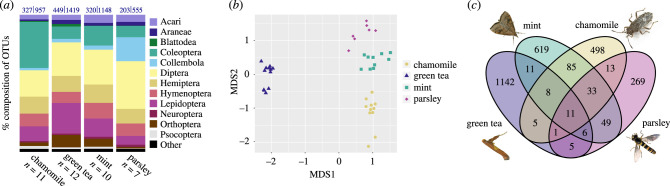
Taxonomic composition of arthropods recovered from dried plant samples. (*a*) Bar plot showing per cent composition of OTUs by arthropod order. Numbers above bars indicate mean number of OTUs|total number of OTUs recovered from each plant. (*b*) NMDS ordination of arthropod composition by sample. (*c*) Venn diagram showing total numbers of OTUs (greater than 10 reads for a given plant) recovered for each plant. Exemplary arthropods are shown next to plants from which they were recovered, clockwise from upper left: *Udea profundalis* (crambid pest of mint), *Nysius senecionis* (lygaeid pest of chamomile), *Sphaerophoria scripta* (hoverfly associated with Apiaceae), *Caloptilia theivora* (gracillariid pest of tea).

The 40 samples for marker set A yielded an average of 32 287 arthropod reads per sample. Altogether, we recovered 3264 arthropod OTUs representing three classes, 22 orders, 281 families, 1068 genera and 1279 species, comprising herbivores, predators, parasitoids and detritivores (figure 2*a*, electronic supplementary material, table S1). Each separate sample recovered more than 200 OTUs on average, with green tea showing the highest mean OTU number (449; figure 2*a*). The order composition was comparable between different plant species (figure 2*a*), with the exception of significantly more coleopteran OTUs in chamomile and collembolan OTUs in parsley (Fisher's exact test, *p* < 0.05). Many taxa were exclusively detected from their host plant (electronic supplementary material, table S1). In NMDS ordination, arthropod taxa from different tea producers cluster by host plant (figure 2*b*). A Venn diagram shows very little overlap in OTU composition between plant species (figure 2*c*). Though the majority of OTUs likely originated from fields before harvest, we also detected a smaller number of OTUs representing typical storage pests, which likely entered the samples after processing and drying (electronic supplementary material, figure S1B). Many of the identified species could be assigned to biogeographic regions (electronic supplementary material, figure S1A, and table S1). For example, numerous Asian species were recovered from green tea (electronic supplementary material, figure S1A, table S1).

## 4. Discussion

Insect monitoring is commonly performed using passive sampling [2]. Our work suggests that eDNA from dried plants is a useful complement to traditional monitoring, providing critical information on plant–arthropod interactions. The recovery of typical pest species for the different herbs and the ecological diversity of recovered taxa underlines the accuracy of our approach. Leaf material can be easily collected in paper bags and dried in the field using silica gel, or in the laboratory by freeze-drying or using drying ovens at moderate temperature. The subsequent grinding in a mortar or blender is also straightforward [27]. Arthropod DNA in dry plant material appears to be very temporally stable: indeed, we found DNA from true plant-associated arthropods to be dominant over that of storage pests, which would have entered the sample later. This is a considerable advantage over eDNA from external plant washes, which is very short-lived [8]. Follow-up work should explore whether decade old stored dried plant samples like herbarium specimens could also be suitable for recovering arthropod DNA.

Our data also reveal the geographical origins of plant samples. For example, many arthropods from mint tea originate in the Pacific Northwest of America, a major peppermint growing area, while typical East Asian species are only found in green tea. Although we performed relatively coarse geographical assignments, haplotype-level information [28] could be used to perform a finer-scaled geographical assignment for widely distributed species, for example to classify a European species as Iberian, Italian or from the Balkans [29]. This could be used for regulatory purposes, e.g. to trace the origin of illegal plant material confiscated by customs [30].

Besides conservation-driven biomonitoring, dried plant material is well suited for pest management. Many pest species live cryptically on or inside their host plant, making them hard to detect. Invasive pests are often only recognized when they have reached very large population sizes [31]. Regularly sequencing dried plant samples would enable detection of such cryptic pests long before outbreaks, or allow the detection of storage pests in warehouses.

The simplicity of collecting and drying plant material makes this an appealing way for laypeople to collect arthropod samples. In combination with the ability to reconstruct diverse arthropod communities from common household items like tea bags, this gives the approach of a ‘bug in a teacup’ high potential for outreach. Dried plant material could serve as an easily acquired terrestrial eDNA matrix to raise awareness of arthropod biodiversity. The collection and storage of dried plant material is straightforward, does not entail killing large numbers of insects, and requires no hazardous chemicals or freezing. This makes the approach ideal for working with schoolchildren.

While our eDNA approach represents an important development for arthropod monitoring, it should be noted that it is not free of biases and will require further standardization in the future. It remains to be tested whether the analysis misses certain taxa, for example because they deposit less eDNA on the plant. Also, the sources of detected arthropod DNA remain to be explored. While part of this DNA may fit the classic definition of eDNA, originating for example from bite marks or faeces, there is likely a substantial contribution of whole specimens of very small taxa or eggs. Our results suggest that a mix of these eDNA sources is present in our data.

## Data Availability

Raw reads as well as OTU tables and alignment used for primer design as well as a README file description for the data are available in the Dryad Digital Repository: https://doi.org/10.5061/dryad.7wm37pvvx [32]. The data are provided in electronic supplementary material [33].
